# Antagonism of the Muscarinic Acetylcholine Type 1 Receptor Enhances Mitochondrial Membrane Potential and Expression of Respiratory Chain Components via AMPK in Human Neuroblastoma SH-SY5Y Cells and Primary Neurons

**DOI:** 10.1007/s12035-022-03003-1

**Published:** 2022-08-25

**Authors:** Farhana Naznin, T. M. Zaved Waise, Paul Fernyhough

**Affiliations:** 1 Division of Neurodegenerative Disorders, St Boniface Hospital Albrechtsen Research Centre, R4046 - 351 Taché Ave, Winnipeg, MB R2H 2A6 Canada; 2 Department of Pharmacology and Therapeutics, University of Manitoba, Winnipeg, MB Canada

**Keywords:** Axon, Diabetic neuropathy, Dorsal root ganglia, Mitochondria, OXPHOS, Plasma membrane potential

## Abstract

Impairments in mitochondrial physiology play a role in the progression of multiple neurodegenerative conditions, including peripheral neuropathy in diabetes. Blockade of muscarinic acetylcholine type 1 receptor (M_1_R) with specific/selective antagonists prevented mitochondrial dysfunction and reversed nerve degeneration in in vitro and in vivo models of peripheral neuropathy. Specifically, in type 1 and type 2 models of diabetes, inhibition of M_1_R using pirenzepine or muscarinic toxin 7 (MT7) induced AMP-activated protein kinase (AMPK) activity in dorsal root ganglia (DRG) and prevented sensory abnormalities and distal nerve fiber loss. The human neuroblastoma SH-SY5Y cell line has been extensively used as an in vitro model system to study mechanisms of neurodegeneration in DRG neurons and other neuronal sub-types. Here, we tested the hypothesis that pirenzepine or MT7 enhance AMPK activity and via this pathway augment mitochondrial function in SH-SY5Y cells. M_1_R expression was confirmed by utilizing a fluorescent dye, ATTO590-labeled MT7, that exhibits great specificity for this receptor. M_1_R antagonist treatment in SH-SY5Y culture increased AMPK phosphorylation and mitochondrial protein expression (OXPHOS). Mitochondrial membrane potential (MMP) was augmented in pirenzepine and MT7 treated cultured SH-SY5Y cells and DRG neurons. Compound C or AMPK-specific siRNA suppressed pirenzepine or MT7-induced elevation of OXPHOS expression and MMP. Moreover, muscarinic antagonists induced hyperpolarization by activating the M-current and, thus, suppressed neuronal excitability. These results reveal that negative regulation of this M_1_R-dependent pathway could represent a potential therapeutic target to elevate AMPK activity, enhance mitochondrial function, suppress neuropathic pain, and enhance nerve repair in peripheral neuropathy.

**Supplementary Information:**

The online version contains supplementary material available at 10.1007/s12035-022-03003-1.

## Introduction

Muscarinic acetylcholine receptors (mAChRs) are members of the superfamily of G protein coupled receptors (GPCRs) and consist of five molecular subtypes (M1–M5) [1, 2]. These receptors are coupled to various signal transduction pathways where M1, M3, and M5 couple with Gq to activate the inositol triphosphate (IP_3_) pathway, and the M2 and M4 receptors couple with Gi to inhibit adenylyl cyclase [3, 4]. The muscarinic acetylcholine type 1 receptor (M_1_R) regulates numerous fundamental functions of the central and peripheral nervous systems and has been targeted for the development of new therapeutic modalities and drugs [5–7]. A variety of molecules block M_1_R activation including pirenzepine, which is a selective orthosteric receptor antagonist with high affinity [8], and muscarinic toxin 7 (MT7) which is a highly specific antagonist (or negative allosteric modulator) [9, 10].

Mitochondria are highly dynamic, energy generating organelles that are known to concentrate in regions of high energy demand [11, 12] and are densely packed in sensory nerve terminal boutons [13, 14]. Mitochondrial membrane potential (MMP) is a marker of optimal mitochondrial function where mitochondrial depolarization can indicate mitochondrial dysfunction [15–17]. Depolarization and the loss of MMP impacts respiratory chain complexes, which interrupts cellular electron flow and results in ATP depletion [18–21]. Maintenance of MMP is fundamental for the normal performance and survival of cells that have a high-energy requirement [22], such as sensory neurons [23–25]. Mitochondrial dysfunction and any energy deficit can contribute to the pathogenesis of neurodegenerative disease such as diabetic sensory neuropathy [23, 26, 27]. Furthermore, abnormal mitochondrial function correlated with a downregulation of mitochondrial proteins, including components of the respiratory chain complex [23, 26, 28]. Interestingly, blockade of M_1_R with pirenzepine or MT7 prevented mitochondrial dysfunction and reversed nerve degeneration in rodent models of diabetic neuropathy [29, 30].

The energy sensor AMP-activated protein kinase (AMPK)/peroxisome proliferator-activated receptor-γ coactivator α (PGC-1α) signaling pathway is linked to mitochondrial biogenesis and function [31, 32] and impaired AMPK/PGC-1α signaling contributes to the aforementioned mitochondrial dysfunction and development of sensory neuropathy in diabetes [24, 30, 33–35]. Increased AMPK phosphorylation, driven by resveratrol or IGF-1, was associated with protection from neuropathy mediated via upregulation of respiratory chain components, augmentation of mitochondrial function, and respiratory complex activities [24, 36, 37]. We have recently shown in adult sensory neurons that pirenzepine and MT7 drive phosphorylation of AMPK mediated via Ca^2+^ influx and activation of Ca^2+^/calmodulin-dependent protein kinase kinase β (CaMKKβ) [29, 30]. This resulted in augmentation of mitochondrial function and elevated neurite outgrowth [29, 30, 38].

Neuronal hyperexcitability is a feature of neuropathic pain. The opening of potassium (K^+^) channels leads to hyperpolarization of the cell membrane which results in a decrease in cell excitability. K^+^ channels, primarily Kv7.2/7.3 sub-types (termed M channels), regulate neuronal excitability in peripheral neurons and are modulated by a large array of receptor types [39–41]. The M-current (I_M_) is sensitive to the M_1_R agonist muscarine [42]. Muscarinic activation of M_1_R mobilizes internal Ca^2+^ stores leading to closure of M channels and inducing a slow and long-lasting depolarization by inhibiting I_M_ and this effect is usually accompanied by a decrease in membrane conductance [43, 44]. This muscarinic suppression of I_M_ was antagonized by pirenzepine [45] through enhancing the I_M_ current to make the neuron less excitable. However, the mechanistic interactions between antimuscarinic drug, mitochondrial membrane potential, and M-current remain to be defined.

Therefore, to advance understanding of the downstream consequences of M_1_R antagonism, we tested the hypothesis that M_1_R antagonism enhances mitochondrial function via activation of the AMPK signaling pathway as well as modulating neuronal excitability in human cells. This comprehensive study aimed at evaluating mitochondrial parameters including oxygen consumption rate (OCR), mitochondrial membrane potential (MMP), and expression of component proteins of the mitochondrial complexes (OXPHOS). We also investigated changes in plasma membrane potential in response to pirenzepine or MT7 in human neuroblastoma SH-SY5Y cells and primary neurons.

## Materials and Methods

### Animals and Cell Culture

The human neuroblastoma SH-SY5Y cell line (ATCC CRL-2266, Virginia, USA) was a kind gift from Dr. Jun-Feng Wang, University of Manitoba. The cells were cultured in DMEM/F12 (1:1) media supplemented with heat inactivated 10% FBS and 1X antibiotic antimycotic solution (A5955, Sigma, St. Louis, MO, USA).

Dorsal root ganglia (DRG) from adult male Sprague–Dawley rats were dissected and dissociated using previously described methods [29]. All animal procedures followed the guidelines of the University of Manitoba Animal Care Committee using the Canadian Committee on Animal Care (CCAC) rules. Neurons were cultured in defined Hams F12 media containing 10 mM D-glucose (N4888, Sigma) supplemented with modified Bottenstein’s N2 additives (0.1 mg/ml transferrin, 20 nM progesterone, 100 mM putrescine, 30 nM sodium selenite, 0.1 mg/ml BSA; all additives were from Sigma). In all experiments, the media was also supplemented with 0.146 g/L L-glutamine, a low-dose cocktail of neurotrophic factors (0.1 ng/ml NGF, 1.0 ng/ml GDNF and 0.1 ng/ml NT-3; all from Promega, Madison, WI, USA), 0.1 nM insulin, and 1X antibiotic antimycotic solution. Cultures were treated with 100 nM MT7 (M-200, Alomone Labs, Jerusalem, Israel) or 1 µM pirenzepine (P7412, Sigma).

### Localization of M_1_R

Fluorescent dye ATTO Fluor 590-conjugated MT7 (MT7-ATTO590; Alomone Labs) was used to detect M_1_R. The activity of this MT7-ATTO590 conjugate on M1R was confirmed by the company (Alomone Labs) in M_1_R/C6 cells by measuring intracellular changes in Ca^2+^ levels and the specific binding was determined in rat DRG culture in the presence of excess (1 µM) unlabeled MT7 (data not shown). Adult wild-type and M_1_R-KO (C57BL/6 background, line 1784; Taconic Biosciences Inc.) [46] mouse DRG tissues were incubated with 100 nM MT7-ATTO590 containing media at 37 °C CO_2_ incubator overnight and then fixed in 2% PFA, cryoprotected in 20% sucrose, and embedded in Tissue-Tek O.C.T. compound to prepare 7 µm sections. All sections were incubated overnight at 4 °C with β-tubulin III antiserum (1:500; T8578, Sigma) and then stained for 1 h with Alexa Fluor 488-conjugated anti-mouse IgG (1:1000; Invitrogen, California, USA) at room temperature. To confirm M_1_R expression in SH-SY5Y cells and cultured rat DRG neurons, cells/neurons were incubated with 100 nM MT7-ATTO590 at 37° C in a CO_2_ incubator overnight for microscopy. All images were taken by using a Carl Zeiss LSM510 confocal or Axioscope-2 fluorescence microscope.

### Small Interfering RNAs (siRNA)-Based Knockdown of AMPK

SH-SY5Y cells were transfected with 10 nM AMPK-specific siRNAs (AMPKα1, cat. 4392420, ID: s100 and s102; AMPKα2, cat. 4390824, ID: s11057, Thermo Scientific, Pittsburgh, PA, USA), or scrambled siRNA (cat. 4390843, Thermo Scientific) using Lipofectamine RNAiMAX (Invitrogen, Life Technologies, USA) according to the instruction manual. Briefly, siRNA was incubated with transfection reagent in Opti MEM (Invitrogen) for 5 min at room temperature to allow the formation of transfection complexes, and then the transfection complexes were added to cells drop-by-drop. Before transfection, the medium was changed to antibiotic-free DMEM. After 24 h of transfection, cells were changed to fresh medium and then subjected to various treatments as required.

### Quantitative Western Blotting

Cell lysate was harvested from cell culture and then homogenized in ice-cold RIPA buffer containing 25 mM Tris pH 8, 150 mM NaCl, 0.1% SDS, 0.5% sodium deoxycholate, 1% Triton X-100, and protease and phosphatase inhibitor cocktail. Protein assay was performed using the DC protein assay (Bio-Rad, CA, USA), and Western blot analysis was conducted. Proteins (15 µg total protein/lane) were resolved and separated via 10% sodium dodecyl sulfate–polyacrylamide gel electrophoresis (SDS-PAGE). The proteins were subsequently transferred to a nitrocellulose membrane (Bio-Rad) using Trans-Blot Turbo Transfer System (Bio-Rad) and immunoblotted with specific antibodies to phosphorylated AMPK (pAMPK on Thr172; 1:1000, Cell Signaling Technology, Massachusetts, USA), total AMPK (T-AMPK; 1:7000, Abcam, Cambridge, UK), total OxPhos (1:1000, Invitrogen; antibody cocktail containing multiple OxPhos antibodies against complex I (20 kDa), complex II (30 kDa), complex III (core 2; 48 kDa), complex IV (MTCO1 subunit, 40 kDa), and complex V (ATP5a subunit, 55 kDa)), NDUFS3 (1:1000, Abcam, complex I, 30 kDa), and total ERK (T-ERK; 1:3000, Santa Cruz Biotechnology, Texas, USA). Of note, total protein bands were captured by chemiluminescent imaging of the blot after gel activation (TGX Stain-Free™ FastCast Acrylamide Solutions, Bio-Rad) in addition to the use of T-ERK levels for target protein normalization (to adjust for loading). The secondary antibodies were HRP-conjugated goat antirabbit IgG (H + L) or goat anti-mouse IgG (H + L) from Jackson ImmunoResearch Laboratories, PA, USA. The blots were incubated in Clarity™ Western ECL substrate (Bio-Rad) or SignalFire™ ECL Reagent (Cell Signaling Technology) and imaged using a Bio-Rad ChemiDoc image analyzer (Bio-Rad).

### Measurement of Mitochondrial Membrane Potential (MMP)

The MMP was evaluated by use of the fluorescent, lipophilic, and cationic probe, 5,5′,6,6′-tetrachloro-1,1′,3,3′-iodide (JC-1) (Invitrogen) according to the manufacturer’s instructions. JC-1 dye stains mitochondria in a membrane potential-dependent manner. In functioning mitochondria with intact membrane potential differential, the mitochondria show a high red-to-green fluorescence ratio, whereas in depolarized mitochondria, the cationic dye is in monomeric form and produces a low red-to-green ratio [47]. The ratio of aggregate (red) to monomer (green) is decreased after the addition of FCCP (an uncoupler that dissipates the transmembrane electrochemical gradient). Cultured SH-SY5Y cells in 96-well plates (black clear-bottomed; Thermo Scientific) were loaded with 20 μM JC-1 and DRG neurons were loaded with 5 μM JC-1 staining solution for 15 min at 37 °C and washed with JC-1 staining buffer and then subjected to various treatments. The fluorescence intensity was measured by a Biotek Synergy Neo2 multimode plate reader with 485 nm for excitation and 530 nm for emission of green (monomer form) fluorescence, and 485 nm for excitation and 590 nm for emission for red (aggregate form) fluorescence. The MMP of cells in each group was evaluated as the fluorescence ratio of red to green. The data were expressed as the relative expression to the control.

### Assessment of Plasma Membrane Potential

Cultured DRG neurons or SH-SY5Y cells in 96-well plates (black clear-bottomed; Thermo Scientific) were loaded with 5 μM of DiBAC4(3) (Invitrogen) staining solution for 30 min at 37 °C to ensure dye distribution across the plasma membrane. DiBAC4(3) is an anionic potentiometric probe that partitions between cells and extracellular solution in a membrane potential-dependent manner [48]. With increasing membrane potential, the probe partitions into the cell, resulting in an increase in fluorescence due to dye interaction with intracellular lipids and proteins, whereas hyperpolarization evokes a decrease in fluorescence. Fluorescence signals were recorded (with a Carl Zeiss LSM510 confocal inverted microscope; excitation at 488 nm and emission 520 nm) for 8 min at 5 s intervals. After measurement of 1 min basal fluorescence, drugs (MT7 100 nM, pirenzepine (PZ) 30 µM, muscarine (Mus) 100 µM; prepared in the assay buffer containing DiBAC4(3)) were administered to the culture. At the end, 90 mM KCl was applied. Only neurons that responded to KCl with membrane depolarization were selected for analysis. Images were analyzed using Fiji software [49]. Regions of interest (ROIs) containing individual neurons were selected and fluorescence intensities quantified. Responses were corrected for any background changes in fluorescence and data were plotted with baseline correction.

### Statistical Analysis

Data are expressed as mean ± SEM, and where appropriate, data were subjected to unpaired 2-tailed Student’s *t* test, one-way ANOVA with Tukey’s, or Dunnett’s multiple comparison post hoc tests. Area under the curve (AUC) analysis was performed using the trapezoidal rule with baseline correction. A value of *p* < 0.05 was considered statistically significant. GraphPad Prism software was used to perform statistical analysis.

## Results

### Expression of M_1_R in Human Neuroblastoma SH-SY5Y Cell Line and Rodent DRG Neurons

**Fig. 1:**
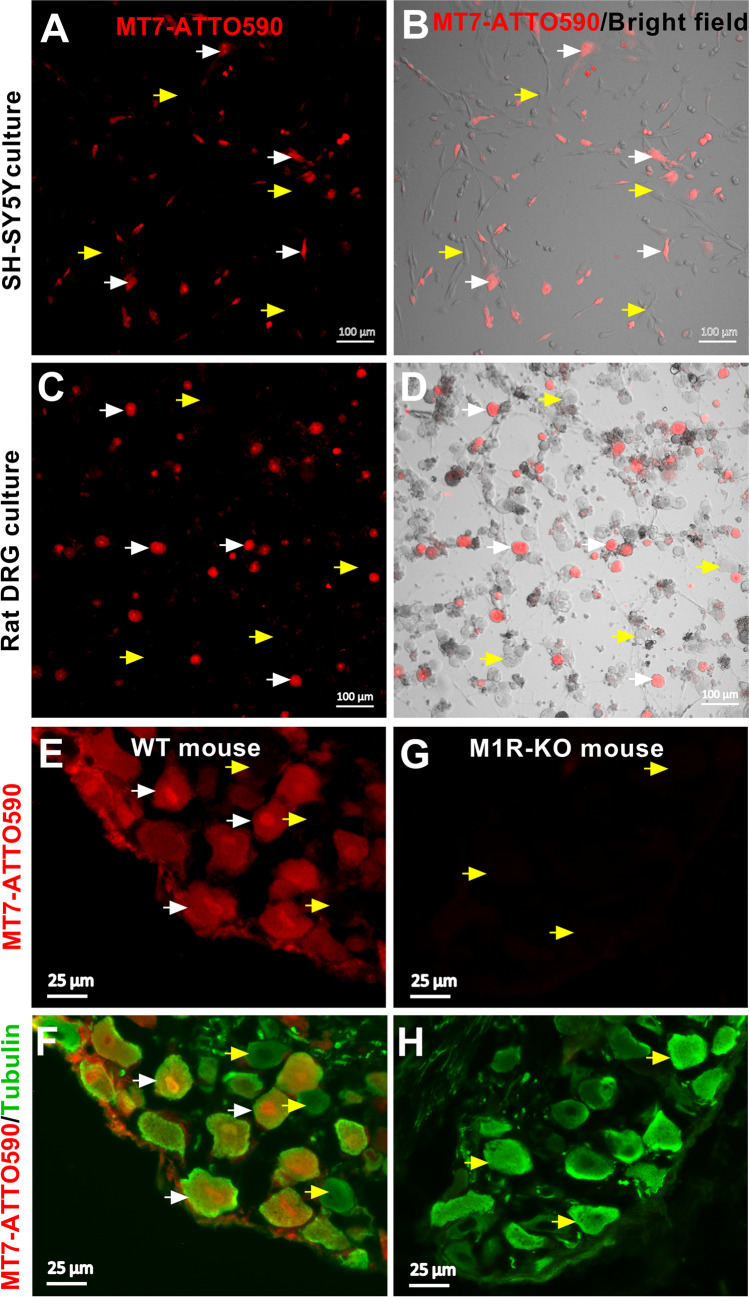
Human neuroblastoma SH-SY5Y cell line and DRG neurons express M1 receptors. **A**–**D** Confocal images of cultured SH-SY5Y cells (**A**, **B**) and rat DRG neurons (**C**, **D**) stained with 100 nM MT7-ATTO590. **E**–**H** Immunohistochemistry images of mouse DRG tissues for wild type (**E**, **F**) and M_1_R KO (**G**, **H**) mice stained with 100 nM MT7-ATTO590. **F, H** Neuronal cells were stained with ß-tubulin III antibodies. White arrows indicate M_1_R + ve and yellow arrows indicate M_1_R -ve cells or neurons. WT, wild type; M_1_R KO, M_1_R knock out

The presence of M_1_R in SH-SY5Y cell line and rat DRG neurons was assessed by using MT7-ATTO590 (Fig. 1A–D). This labelled MT7 is absolutely specific for the M_1_R and is superior to the use of antibodies that cross-react with other MR sub-types. The specificity of MT7-ATTO590 was confirmed by using DRG tissues from wild-type and M_1_R knock out (M_1_R KO) mice (Fig. 1E–H). The SH-SY5Y human neuroblastoma cell line is a well-characterized model to study muscarinic cholinergic function [50, 51] and we decided to use this cellular model to establish the effect of M_1_R antagonism. We also confirmed the mRNA expression levels of M_1_R in SH-SY5Y cells by using quantitative RT-PCR (Supplementary Fig. 1) and this data confirmed a previous report [52]. Overall, these observations clearly demonstrate that M_1_R is widely expressed in rodent DRG and neuroblastoma cells.

### Pirenzepine/MT7 Augment AMPK Phosphorylation in a Dose- and Time-Dependent Manner and Enhance Respiratory Chain Protein Expression and Mitochondrial Function in SH-SY5Y Cells

**Fig. 2:**
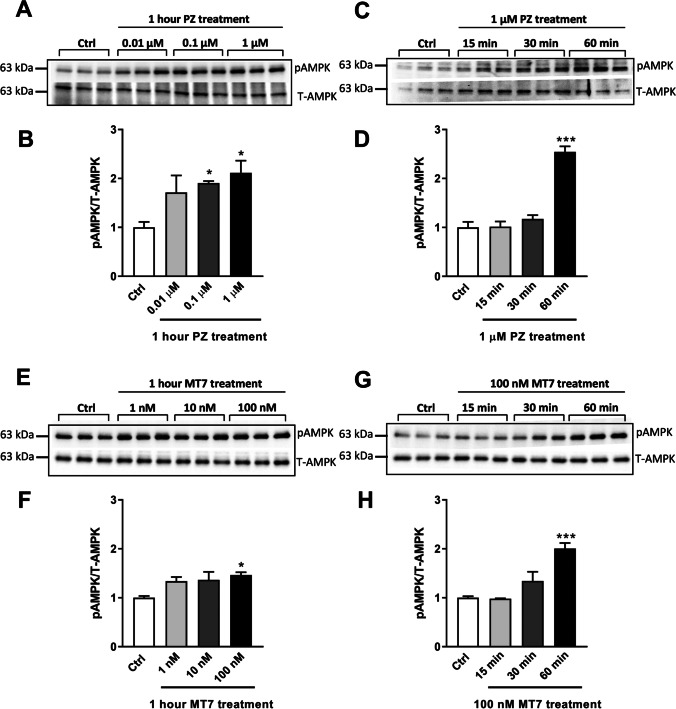
Pirenzepine and MT7 elevate phosphorylation of AMPK in a dose and time-dependent manner. **A** SH-SY5Y cells were cultured overnight, serum deprived (SD) for 4 h and then treated with various doses of pirenzepine (PZ) for 1 h. Western blots are shown for P-AMPK and T-AMPK. **B** Levels of expression of P-AMPK (in A) presented relative to T-AMPK. **C** SH-SY5Y cells were subjected to SD exposed to 1 µM PZ for various times (15 min, 30 min, and 60 min). **D** Levels of P-AMPK (in C) presented relative to T-AMPK. **E** SH-SY5Y cells were cultured overnight, starved for 4 h and then treated with various doses of MT7 for 1 h. Western blots are shown for P-AMPK and T-AMPK. **F** Levels of expression of P-AMPK (in E) presented relative to T-AMPK. **G** SH-SY5Y cells were subjected to SD exposed to 100 nM MT7 for various times (15 min, 30 min, and 60 min). **H** Levels of P-AMPK (in G) presented relative to T-AMPK. Data are expressed as mean ± SEM, *n* = 3 replicates; *p < 0.05 and ***p < 0.001 vs control by one-way ANOVA with Dunnett’s post hoc test

**Fig. 3:**
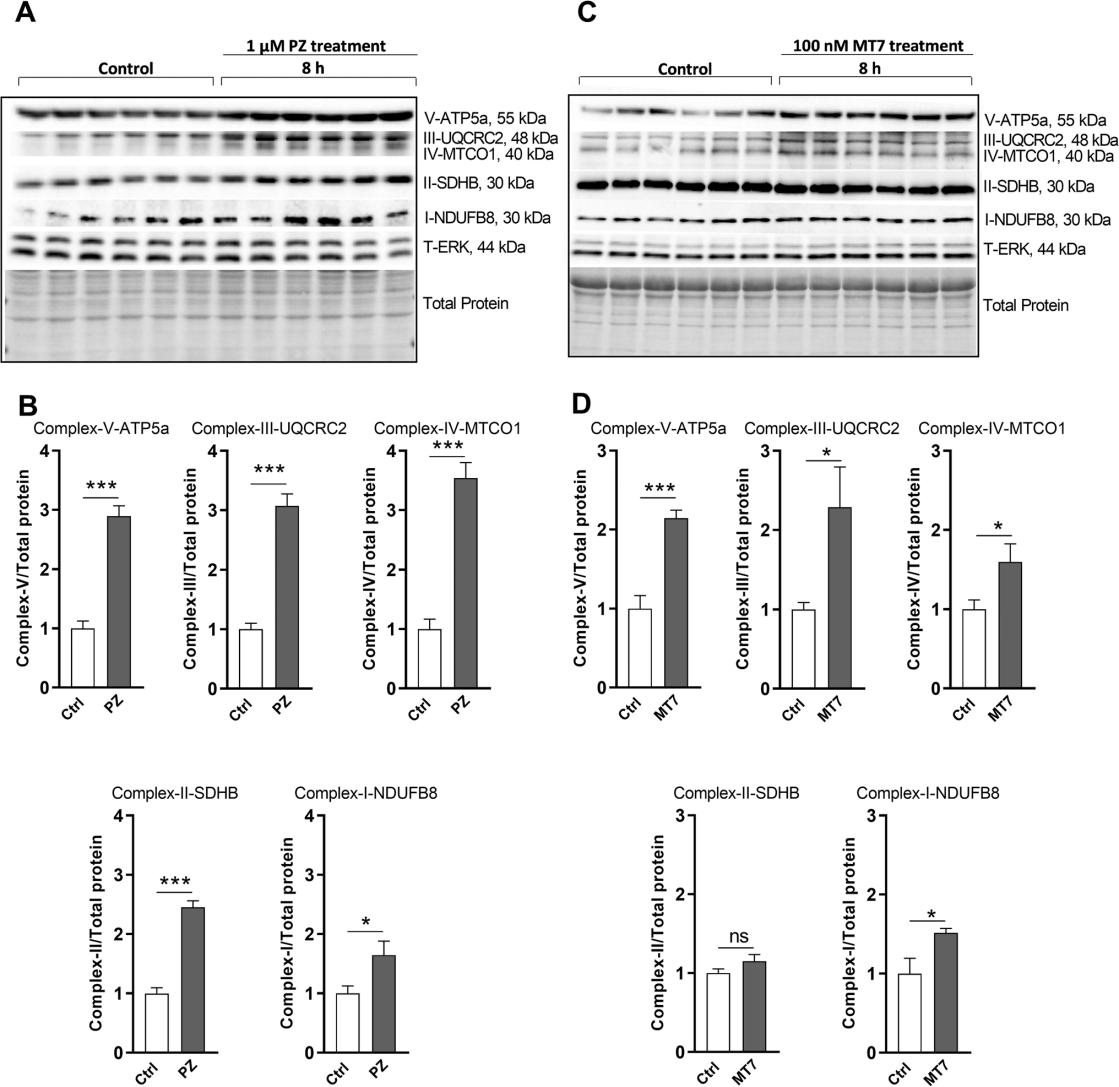
Pirenzepine and MT7 treatment increase the expression of mitochondrial respiratory chain proteins. **A**–**D** SH-SY5Y cells were treated with/without 1 µM pirenzepine (PZ; A, B) and 100 nM MT7 (C, D) for 8 h, and lysates subjected to Western blotting. Representative Western blot (A, C) showing OXPHOS protein levels. Specific proteins from each respiratory complex were quantified and expressed relative to total protein (B, D). Data are expressed as mean ± SEM, *n* = 6 replicates; *p < 0.05 or **p < 0.01 or ***p < 0.001 vs control by unpaired Student’s *t*-test

Pirenzepine- or MT7-induced AMPK activation was confirmed by detecting the phosphorylated form of AMPK (Fig. 2). SH-SY5Y cells were starved in serum free DMEM for 4 h before treatment. Western blots exhibited a marked dose-dependent and time-dependent elevation in AMPK phosphorylation (pAMPK) following pirenzepine/MT7 treatment. Quantification of pAMPK relative to T-AMPK revealed ~ 2.0-fold elevation at 1 µM PZ (Fig. 2A, B) and ~ 1.5-fold elevation at 100 nM MT7 (Fig. 2E, F). A time course for the effect of 1 µM pirenzepine and 100 nM MT7 was performed and revealed an elevation in pAMPK levels following 1 h of treatment, where pirenzepine caused a ~ 2.5-fold increase (Fig. 2C, D) and MT7 caused a ~ 2.0-fold enhancement in pAMPK (Fig. 2G, H). Previous studies reported that there are several downstream effectors of AMPK that contribute to the regulation of mitochondrial biogenesis [53, 54]. In line with these observations, pirenzepine/MT7 treatment (8 h, without starvation) induced mitochondrial OXPHOS proteins, components of the electron transport chain (ETC), including complex components V-ATP5a, III-UQCRC2, IV-MTCO1, II-SDHB, and I-NDUFB8 (Fig. 3A-D). In serum-deprived condition, 8 h treatment with pirenzepine or MT7 also exhibited enhanced mitochondrial protein expression (Supplementary Fig. 2), although some protein complexes were not statistically significant and changes not as robust as compared with the data (without starvation) revealed in Fig. 3. This may be explained by the fact that cells were subjected to increasing duration of serum deprivation and so experience a stressful condition [55]. In addition to mitochondrial protein expression, mitochondrial oxygen consumption rate (OCR) was enhanced with M_1_R antagonist treatment (Supplementary Fig. 3). The bioenergetic parameter of maximal respiration was also increased, although not reaching statistical significance (*P* < 0.06) (Supplementary Fig. 3C). Relative ATP production, measured using the Seahorse machine, was augmented by MT7 treatment (Supplementary Fig. 3E). This confirms our previous work in cultured rat DRG neurons, and in tissues from STZ-induced diabetic rodents [29]), that blockade of M_1_R enhances mitochondrial function.

### AMPK Knockdown Blocks Upregulation of Mitochondrial Respiratory Protein Complexes Driven by M_1_R Antagonists Pirenzepine and MT7 in SH-SY5Y Cells

**Fig. 4:**
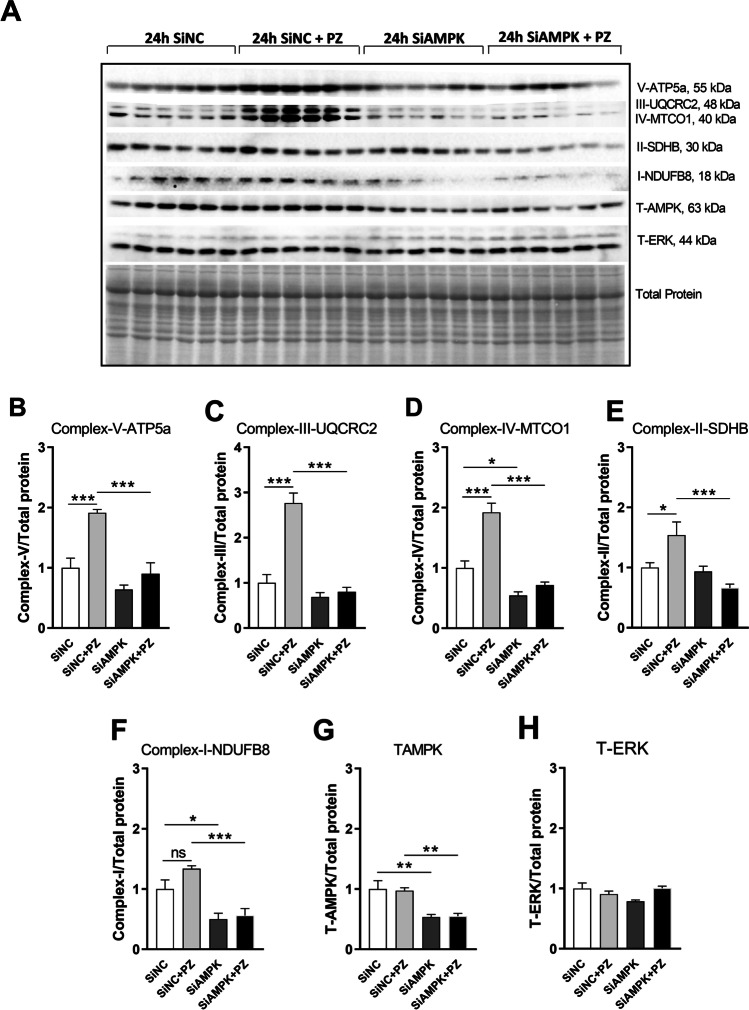
AMPK knockdown blocks pirenzepine-mediated upregulation of mitochondrial respiratory protein complexes. SH-SY5Y cells were cultured overnight, transfected with scrambled siRNA (siNC) or siRNAs specific to AMPK-isoforms α1 and α2 (siAMPK) and cultured for 24 h. Cells were subsequently treated with/without 1 µM PZ for 8 h, and subjected to Western blotting. **A** Representative Western blot showing OXPHOS protein levels for control + siNC, PZ + siNC, control + siAMPK, and PZ + siAMPK. **B**–**G** Band intensity of each protein was normalized to total protein. Western blotting for total AMPK was used to calculate the knock-down efficiency of AMPK isoforms (A) where levels of expression of T-AMPK are presented relative to total protein (G). **H** Total ERK (T-ERK) was used as a loading control. Data are expressed as mean ± SEM, *n* = 6 replicates; *p < 0.05 or **p < 0.01 or ***p < 0.001 by one-way ANOVA with Tukey’s post hoc test

**Fig. 5:**
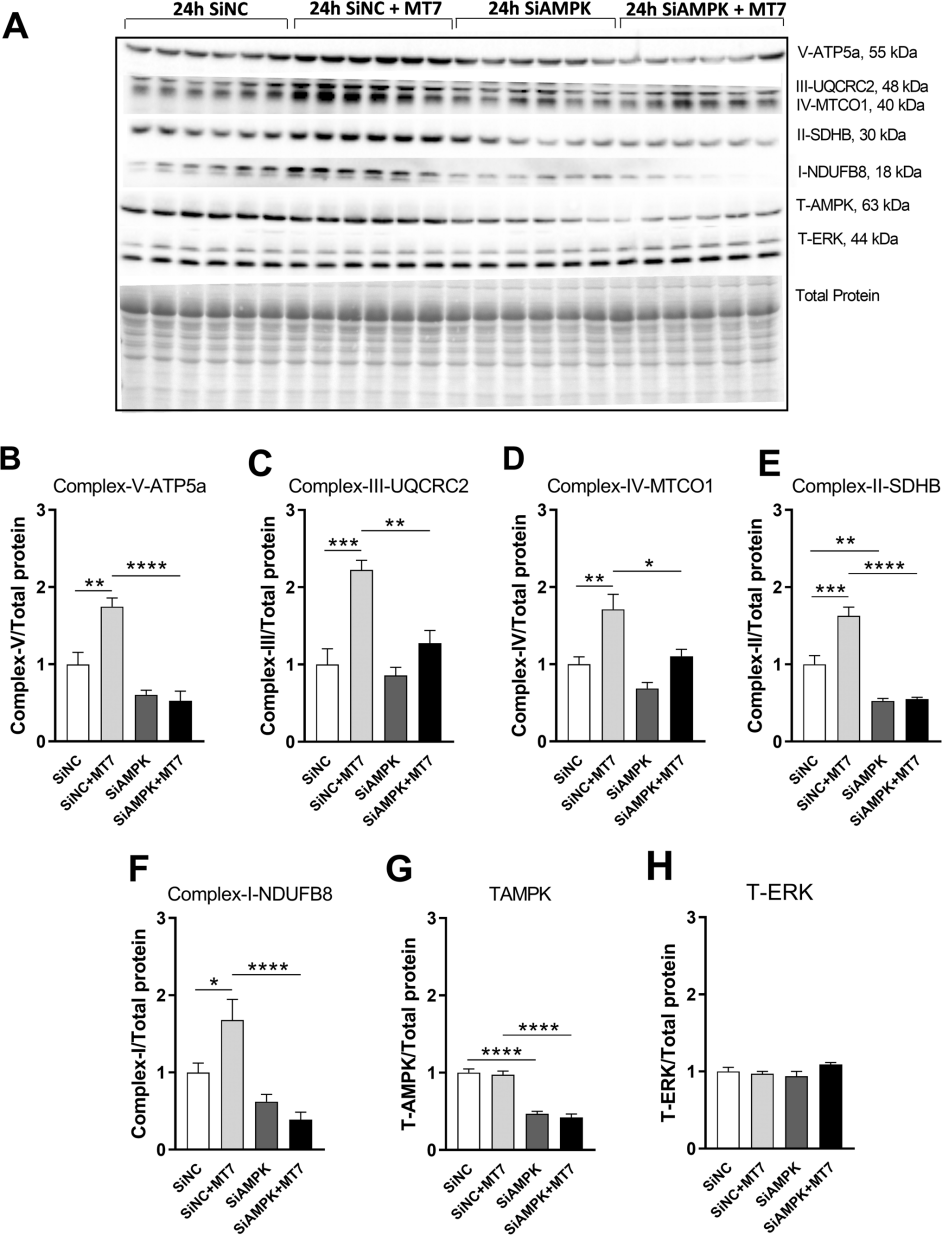
AMPK knockdown blocks MT7-dependent upregulation of mitochondrial respiratory protein complexes. SH-SY5Y cells were cultured overnight, transfected with scrambled siRNA (siNC) or siRNAs specific to AMPK-isoforms α1 and α2 (siAMPK) and cultured for 24 h. Cells were subsequently treated with/without 100 nM MT7 for 8 h, and subjected to Western blotting. **A** Representative Western blot showing OXPHOS protein levels for control + siNC, MT7 + siNC, control + siAMPK, and MT7 + siAMPK. **B**–**G** Band intensity of each protein was normalized to total protein. Western blotting for total AMPK was used to calculate the knock-down efficiency of AMPK isoforms (A) where levels of expression of T-AMPK are presented relative to total protein (G). **H** T-ERK was used as a loading control. Data are expressed as mean ± SEM, *n* = 6 replicates; *p < 0.05 or **p < 0.01 or ***p < 0.001 by one-way ANOVA with Tukey’s post hoc test

Impaired AMPK signaling in DRG neurons is linked to mitochondrial dysfunction [24]. To confirm the causal involvement of AMPK activation in upregulation of mitochondrial protein complexes by pirenzepine/MT7, we employed siRNA-mediated AMPK knockdown in SH-SY5Y cells. Following 24 h of treatment with the siRNAs, the level of total-AMPK protein was significantly depleted (Figs. 4G and 5G). AMPK knockdown significantly blocked the upregulation of mitochondrial OXPHOS proteins induced by pirenzepine (Fig. 4A–F) or MT7 treatment (Fig. 5A–F).

### AMPK Inhibition or Downregulation Suppresses the Pirenzepine/MT7 Effect on Mitochondrial Membrane Potential (MMP) in SH-SY5Y Cells and Rat DRG Neurons

**Fig. 6:**
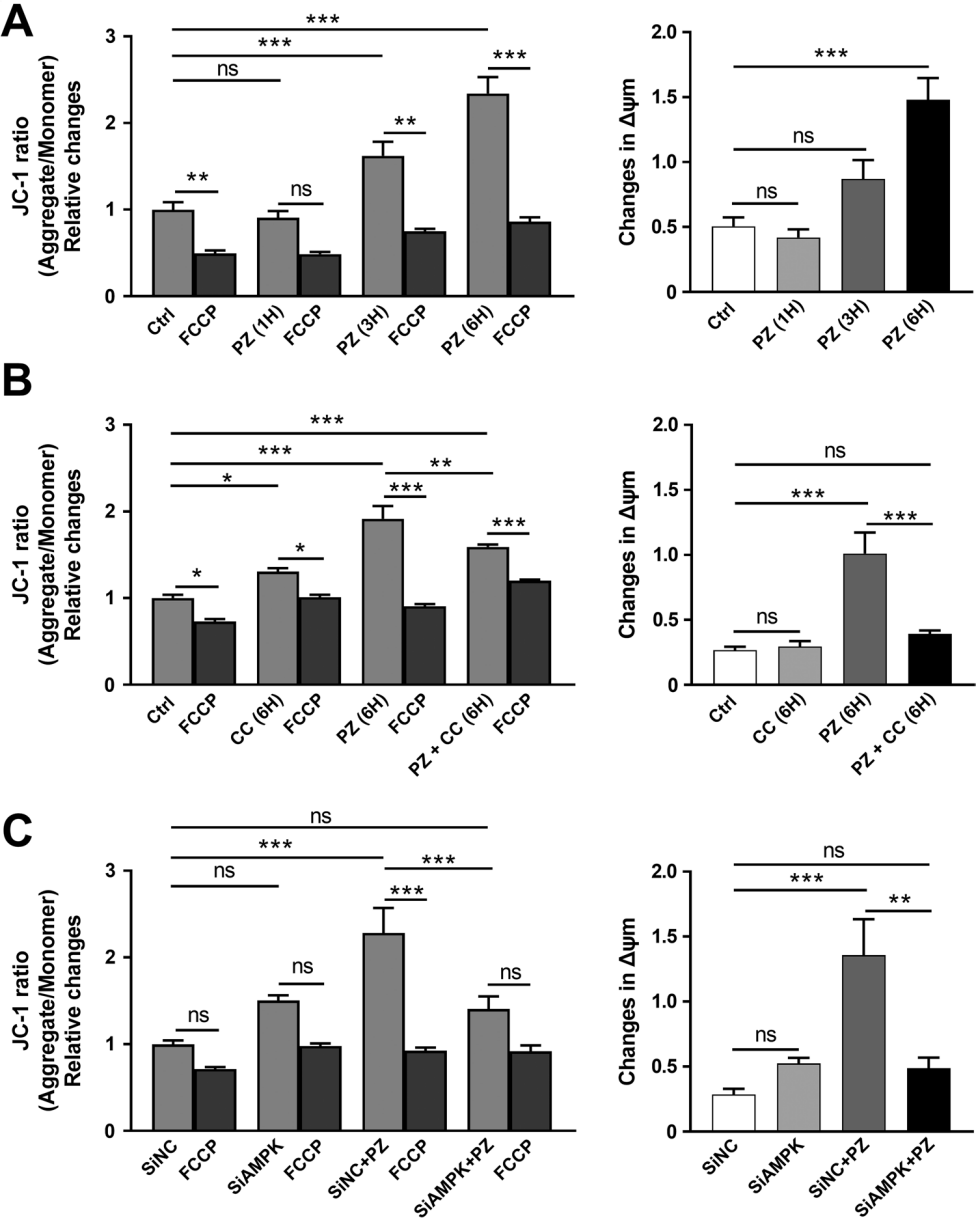
Effects of pirenzepine treatment on mitochondrial membrane potential in SH-SY5Y cells. SH-SY5Y cells were cultured overnight, stained with JC-1 dye to analyze mitochondrial membrane potential (MMP) and subsequently, the loss of MMP in response to FCCP. **A** Cells were treated with/without 1 µM pirenzepine (PZ) for various times (1 h, 3 h, and 6 h). **B** Cells were treated with AMPK inhibitor compound C (CC, 3 µM) with/without 1 µM PZ for 6 h. **C** SH-SY5Y cells were cultured overnight, transfected with scrambled siRNA (siNC) and siRNAs specific to AMPK-isoforms α1 and α2 (siAMPK) and were cultured for 24 h. Cells were subsequently treated with/without 1 µM PZ for 6 h. Fluorescence ratio was used for MMP quantitative analysis. The ratio of aggregate to monomer is decreased after the addition of FCCP (an uncoupler). All the left panels show the MMP, whereas the right panels show changes in MMP after FCCP treatment. The JC-1 dye ratio was determined using a Biotek Neo2 Synergy multimode plate reader. Data are expressed as mean ± SEM, *n* = 10–15 replicates; *p < 0.05 or **p < 0.01 or ***p < 0.001 by one-way ANOVA with Tukey’s post hoc test

**Fig. 7:**
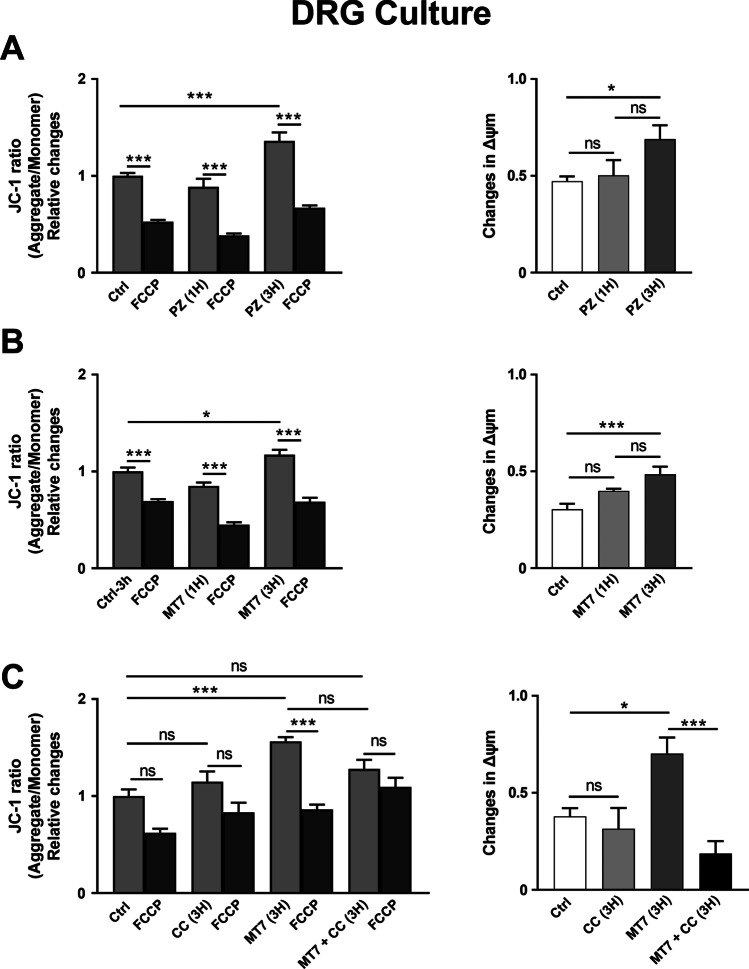
Effects of pirenzepine and MT7 treatment on MMP in DRG neurons. DRG neurons derived from adult control rats were cultured for 24 h, stained with JC-1 dye to evaluate MMP and the loss of MMP subsequent to FCCP application. **A**, **B** Neurons were treated with/without 1 µM PZ (A) or 100 nM MT7 (B) for various times (1 h and 3 h). **C** Neurons were treated with AMPK inhibitor compound C (CC, 3 µM) with/without 100 nM MT7 for 3 h. All the left panels show the MMP, whereas the right panels show changes in MMP after FCCP treatment. Data are expressed as mean ± SEM, *n* = 8–10 replicates; *p < 0.05 or **p < 0.01 or ***p < 0.001 by one-way ANOVA with Tukey’s post hoc test

MMP generated by the proton pumps of the mitochondrial respiratory complexes is indispensable in the process of energy storage during oxidative phosphorylation [56]. MMP, a key indicator of cell health or injury, has become a useful parameter for monitoring changes in mitochondrial function [57]. Changes in MMP were analyzed by employing the mitochondrial cationic dye, JC-1 (Figs. 6 and 7). A time course experiment for the effect of 1 µM pirenzepine was performed in SH-SY5Y cells. Exposure to pirenzepine for 6 h increased the MMP in SH-SY5Y cells (Fig. 6A). Similar time course experiment for 1 µM pirenzepine and 100 nM MT7 was performed in cultured DRG neurons where MMP was elevated after 3 h of pirenzepine (Fig. 7A) or MT7 treatment (Fig. 7B). To see whether this upregulation was due to AMPK activation, SH-SY5Y cells and DRG neurons were treated with Compound C (a pharmacological AMPK inhibitor) or transfected with siRNAs to AMPK. Pharmacological blockade of AMPK using Compound C suppressed the pirenzepine (Fig. 6B) or MT7 (Fig. 7C) induced elevation of MMP. siRNA-based inhibition of AMPK also exhibited a similar suppression of pirenzepine-induced enhancement of MMP in SH-SY5Y cells (Fig. 6C).

### Role of M_1_R Antagonists in the Regulation of Plasma Membrane Potential in Primary DRG Neurons and SH-SY5Y Cells

**Fig. 8:**
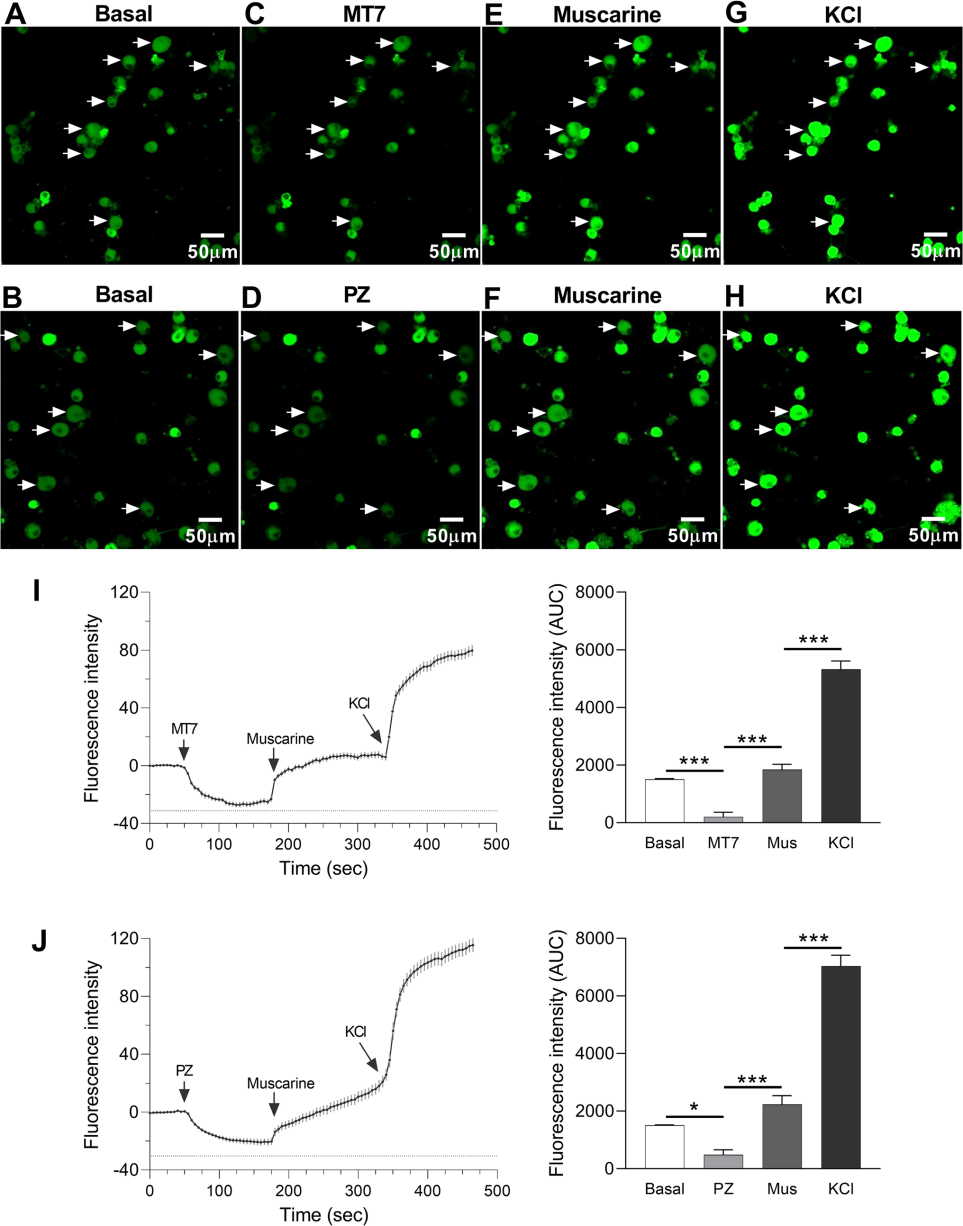
Changes in the plasma membrane potential in response to M_1_R antagonists or agonist in DRG neurons. **A**–**H**. Confocal images of primary cultures of DRG neurons in the presence of the resting membrane potential probe DiBAC4(3) showing fluorescence at basal (A, B) and after administration of 100 nM MT7 (C), 30 µM PZ (D), 100 µM muscarine (Mus; E, F), and 90 mM KCl (G, H). Arrows indicate a selection of neurons that responded to MT7 or PZ. **I–J**. Traces of DiBAC4(3) fluorescence intensity (left panel) and AUC (right panel) show the changes in plasma membrane potential measured in response to MT7 (I) or PZ (J) followed by muscarine and KCl. The AUC was estimated for 1 min before each treatment (MT7/PZ, Mus, KCl) from the baseline to a relative fluorescence level of − 30. Data are expressed as mean ± SEM, *n* = 50–54 neurons; *p < 0.05 or **p < 0.01 or ***p < 0.001 by one-way ANOVA with Tukey’s post hoc test

Cultured DRG neurons were loaded with the voltage sensor probe DiBAC4(3) to evaluate the plasma membrane potential (Vm). In DRG neurons, M_1_R antagonists (MT7 or pirenzepine) induced hyperpolarization thus inducing a less excitable state. The muscarinic receptor agonist, muscarine, depolarized the neuronal plasma membrane potential (Fig. 8A–J). The same experiment was performed in SH-SY5Y cells where there was a similar hyperpolarizing response to MT7 and depolarization to muscarine (Supplementary Fig. 4A-F).

## Discussion

The findings in our current study indicate a link between impaired AMPK signaling and mitochondrial respiratory chain dysfunction in human neuroblastoma SH-SY5Y cells. The SH-SY5Y cell line has been used extensively as an in vitro model system of peripheral sensory neurons as they exhibit traits of sensory neuron phenotype [58]. Thus, the rationale for this approach is that the assay performed in this cell line can be a predictor of efficacy in human cells and will be useful for future drug screening endeavors [59]. In addition, this work provides important background information that will underpin future molecular studies not feasible in primary neurons, e.g., proteomic studies to understand molecular pharmacology at the M_1_R (for example, see [60]). We observed MT7 and pirenzepine treatment enhanced AMPK phosphorylation, augmented mitochondrial complex protein expression, and enhanced mitochondrial function in the SH-SY5Y cell line, and these data support our previous report in DRG neurons [23, 24, 29, 30]. Importantly, siRNA targeting AMPK significantly blocked the drug-induced upregulation of mitochondrial OXPHOS proteins resulting in a suppressed oxidative phosphorylation system.

Dynamic morphological changes in mitochondria are required to maintain a homogenous population of functional mitochondria to ensure continuous and optimal mitochondrial respiration. Optimal mitochondrial function is a key factor for axonal outgrowth and repair [37, 61]. Mitochondrial abnormalities have been proposed to mediate development of diabetic complications through cellular dysfunction in endothelial cells, skeletal muscle, cardiomyocytes, and neurons [23, 24, 26, 62–64]. Mitochondrial biogenesis is triggered by the AMPK-PGC-1α-Nrf1 pathway which, in turn, regulates the expression of both mitochondrial and nuclear genes encoding respiratory chain subunits and other proteins that are required for mitochondrial function [65, 66]. Energy supplementation provided by this pathway is required for axonal outgrowth and neuronal growth [65]. Previous studies have highlighted that activation of AMPK can elevate neurite outgrowth. For example, resveratrol, an activator of AMPK, drives axonal outgrowth and was protective against diabetic neuropathy in STZ-induced diabetic rats [24, 67]. Recent studies also report IGF-1-mediated upregulation of mitochondrial respiration together with a dose-dependent stimulation of ATP production through AMPK in a type 1 model of diabetes [36]. Other works have determined that certain mitochondrial complexes and mitochondrial membrane potential were impaired in cortical tissues and primary DRG neurons from diabetic rat but the cellular mechanisms are not completely understood [23, 25, 68].

The present study demonstrates for the first time that blockade of the M_1_R by the specific antagonist MT7 or the selective antagonist pirenzepine causes an augmentation of the mitochondrial membrane potential (MMP) in both cultured SH-SY5Y cells and DRG neurons. This stimulatory effect on MMP was time dependent and triggered within 1 h. MMP is a parameter for mitochondrial metabolic state and provides an estimate of the ATP production within individual mitochondria [69]. The AMPK inhibitor, Compound C, abolished the pirenzepine and MT7-mediated upregulation of mitochondrial MMP. SiRNA-based inhibition of endogenous AMPK exhibited a similar suppression of the pirenzepine enhancement of MMP. These novel observations in neurons provide functional evidence linking AMPK and alterations in mitochondrial performance, such as maintenance of MMP.

M_1_R activation inhibits voltage-gated Kv7 potassium channels that mediate the M-current in sympathetic neurons [43, 70]. M-current (I_M_) is a low-threshold, slowly activating potassium current in sympathetic neurons where it functions as a “brake” for neurons receiving persistent excitatory input [70]. The M-current is strongly suppressed by M_1_R activation [42, 45, 71, 72] which is known to play an important role in modulating neuronal excitability and its suppression is predicted to increase input resistance in response to excitatory synaptic inputs [70, 73–75]. M-current inhibition via M_1_R activation by acetylcholine is phosphatidylinositol-4,5-bisphosphate (PIP_2_)-dependent with depletion of PIP_2_ dramatically decreasing Kv7 channel open probability [76, 77]. Acute ACh activation of M_1_R promotes PIP_2_ hydrolysis through phospholipase C activation, resulting in PKC phosphorylation and generation of inositol triphosphate, which induces endoplasmic reticulum Ca^2+^ release [78]. Downstream Ca^2+^-dependent pathways drive closing of Kv7 channels, and the outcome is an enhanced propensity for depolarization of the plasma membrane. Interestingly, activated PKC may also contribute to the muscarinic inhibition of Kv7 channels [79]. Activated PKC phosphorylates the C-terminus in the calmodulin (CaM) binding site of the Kv7.2 subunit assisted by A-kinase-anchoring protein AKAP79/150. The phosphorylated state of the channel destabilizes the Kv7 channel/PIP_2_ complex and consequently PIP_2_ hydrolysis suppresses the M-current [80–83].

Kv7/M-channel activity represents an integral regulator of PNS sensitivity downstream of multiple transduction mechanisms likely to contribute to dampening of peripheral pain pathways [84]. They are densely expressed at the sites of spike generation, e.g., axon initial segment of central neurons and terminals of peripheral nociceptive neurons [85, 86]. Previous investigations of the role of Kv7 in regulating neuronal excitability, pain pathways, and nociceptive behaviors utilized pharmacological M-channel blockers or enhancers [41, 87–90]. M-current perturbations were strongly implicated in neuronal hyperexcitability underlying epilepsy and ALS [87, 91], neuroinflammation [92], and neuropathic pain [93, 94]. M-current “opener” compounds have been suggested to be efficacious in preventing brain damage after multiple types of insults/diseases, such as stroke, traumatic brain injury, drug addiction, and mood disorders [95]. However, sensory neurons express Kv7 channels and exhibit the M-current, activated at near resting potential such that at subthreshold potentials produce a prominent outward current [41, 42, 96] helping to keep the resting potential within a hyperpolarized range but an initiating role of M_1_R in this pathway has not been directly elucidated [41, 97]. In accordance with this concept, the present experiments revealed that antimuscarinic drugs pirenzepine or MT7 have a novel mechanism of action acting as putative positive modulators of Kv7 M-channels, i.e., Kv7 channel opener/enhancers in SH-SY5Y cells and sensory neurons. Consequently, M_1_R antagonists help to establish the neuronal resting membrane potential by providing a continual hyperpolarizing influence and make the neurons less excitable. The effects of pirenzepine/MT7 on M-current activation were reversed by muscarinic agonist muscarine leading to increased responsiveness of neurons toward depolarizing stimuli.

As such, the data presented here offer promising evidence for the pivotal role of the Kv7 channel as a target of M_1_R antagonists to stabilize membrane potential as well as dampening deviations in depolarization and, therefore, preventing ectopic firing and spontaneous pain. Importantly, upon axotomy, sensory neurons exhibit spontaneous electrical activity that consumes extensive ATP [98–101]. M_1_R antagonism enhances neurite outgrowth of axotomized adult sensory neurons in culture. Therefore, enhancement of the M-current would reduce the possibility of depolarization, thus theoretically preserving ATP to support actin treadmilling in the growth cone and enhancing axon outgrowth [29, 102]. Thus, pirenzepine and MT7 could be signaling via two self-supporting but different pathways to drive axon outgrowth: the AMPK pathway, which is dependent upon a drug-induced rise in intracellular Ca^2+^ and activation of CaMKKβ [29, 30], and a supplementary pathway involving antimuscarinic elevation of the M-current and hyperpolarization of the plasma membrane and conservation of ATP levels. This latter pathway would be expected to downregulate AMPK activity; however, we propose the drug-induced Ca^2+^ influx overrides this effect. At this stage, we have no evidence that antimuscarinic drug action, possibly mediated through the opening of Kv7 channels, has any role in AMPK activation. However, recent work localizing functional Kv7.4 channels to the mitochondria of cardiac myocytes and CNS neurons provides an intriguing link between the M-current and regulation of cellular bioenergetics and is worthy of future investigation in adult sensory neurons [103].

## Conclusions

Our present findings highlight the utility of muscarinic receptor antagonism as a tool to manipulate the AMPK pathway which is a central component of the pathogenic cascade linking mitochondrial function with neurodegeneration. We have demonstrated that pirenzepine or MT7 enhances mitochondrial function via AMPK and regulate mitochondrial membrane potential and the plasma membrane potential. Pirenzepine or MT7 enhances the M-current activity that is crucially important for controlling the excitability of neurons. Thus, these findings strengthen the case for using M_1_R antagonists for improvement of mitochondrial function, while the ability to suppress excitability of sensory neurons may offer routes for treatment of neuropathic pain as well as simultaneously promoting nerve regeneration in neurodegenerative diseases.

## Supplementary Information

Below is the link to the electronic supplementary material.

ESM 1 Supplemental Fig. 1. M1 receptor expression in human neuroblastoma SH-SY5Y cells. M_1_R expression detected in SH-SY5Y cells measured by real time qPCR. **Method:** RNA was extracted from cultured SH-SY5Y cells using TRIzol® Reagent (Invitrogen). Complementary DNA (cDNA) was synthesized from RNA samples by using the iScript™ gDNA Clear cDNA Synthesis Kit (Bio-Rad) according to the manufacturer’s instructions. Quantitative real-time PCR (QRT-PCR) was performed using Bright Green Master mix (Abmgood Co., Richmond, Canada) compatible with the iQ5 Cycler machine (Bio-Rad). The mRNA level of 18S was used for normalization. Primer sequences for gene expression analysis are listed as follows: M_1_R (CHRM1)-F: 5′- CGGAACTCTGCAACAACAAAGCCTTCCG -3′, M_1_R (CHRM1)-R: 5′- CTTGCGCCAGCGTCTCTTGT-3′, 18S-F: 5′-GCCGCTAGAGGTGAAATTCTTG-3′, 18S-R: 5′- CATTCTTGGCAAATGCTTTCG-3′. (PNG 215 kb)

High resolution image (TIF 3380 kb)

ESM 2 Supplemental Fig. 2. Pirenzepine and MT7 treatment increase the expression of electron transport chain proteins in serum deprived conditions. **A, B** SH-SY5Y cells were serum deprived and treated with/without 1 μM PZ for 8h and lysates subjected to Western blotting. Starvation and treatment were started at the same time point. Specific proteins from each respiratory complex were quantified and expressed relative to total protein. **C, D** SH-SY5Y cells were serum deprived and treated with/without 100 nM MT7 for 8h and lysates subjected to Western blotting. Data are expressed as mean ± SEM, n = 6 replicates: *p < 0.05 or **p < 0.01 or ***p < 0.001 vs control by unpaired Student’s t-test. PZ, pirenzepine. (PNG 768 kb)

High resolution image (TIF 12549 kb)

ESM 3 Supplemental Fig. 3. M_1_R antagonist upregulates mitochondrial respiration and ATP production in SH-SY5Y cells. **A-E**. Mitochondrial respiration was measured using Seahorse XF24 Analyzer. Data were normalized to protein concentration units per well prior to statistical analysis. SH-SY5Y cells were serum deprived and treated with/without 100 nM MT7 for 1h and 3h. Data are expressed as mean ± SEM, n = 5-7 replicates; *p < 0.05 or **p < 0.01 or ***p < 0.001 by one-way ANOVA with Tukey’s post hoc test. **Method:** An XF24 analyzer (Seahorse Biosciences, Billerica, MA, USA) was used to measure the basal level of mitochondrial oxygen consumption rate (OCR), the maximal respiration, the spare respiratory capacity and the coupling efficiency. In short, SH-SY5Y culture medium was changed 1 h before the assay to unbuffered DMEM (Dulbecco’s modified Eagle’s medium, pH 7.4) supplemented with 1 mM sodium pyruvate, and 5 mM D-glucose. Four mitochondrial complex inhibitors including oligomycin (1 μM), FCCP (4 μM) and rotenone (1 μM) combined with antimycin A (1 μM) were injected sequentially through ports in the Seahorse Flux Pak cartridges. Oligomycin acts as an irreversible ATP synthase inhibitor, FCCP as an uncoupler, rotenone as Complex I inhibitor, and antimycin A as an inhibitor of Complex III of the mitochondrial electron transport system. After OCR measurement, cells were subjected to protein assay (DC protein assay) for normalization purposes. OCR measures from each well were normalized to total protein levels and are presented as pmoles/min/mg protein. (PNG 258 kb)

High resolution image (TIF 2079 kb)

ESM 4 Supplemental Fig. 4. Changes in the plasma membrane potential in response to M_1_R antagonists or agonist in SH-SY5Y cells. **A-D** Confocal images of cultures of SH-SY5Y cells in the presence of the voltage sensor probe DiBAC4(3) showing fluorescence at basal (A) and after administration of 100 nM MT7 (B), 100 μM muscarine (C), and 90 mM KCl (D). Arrows indicate some of the cells that responded to MT7. **E-F**. Traces of DiBAC4(3) fluorescence intensity (E) and AUC (F) showing the changes in plasma membrane potential measured in response to MT7 followed by muscarine and KCl. The AUC was estimated for 1 minute before each treatment (MT7, Mus, KCl) from the baseline to a fluorescence level of -20. Data are expressed as mean ± SEM, n = 38 neurons; *p < 0.05 or **p < 0.01 or ***p < 0.001 by one-way ANOVA with Tukey’s post hoc test. (PNG 898 kb)

High resolution image (TIF 8942 kb)

## Data Availability

The datasets used and/or analyzed during the current study are available from the corresponding author on reasonable request.
