# Supplementary figures and images for: Antagonism of the Muscarinic Acetylcholine Type 1 Receptor Enhances Mitochondrial Membrane Potential and Expression of Respiratory Chain Components via AMPK in Human Neuroblastoma SH-SY5Y Cells and Primary Neurons
Source: Mol Neurobiol. 2022 Aug 25;59(11):6754–70. doi: 10.1007/s12035-022-03003-1 (PMC9525428; doi:10.1007/s12035-022-03003-1)

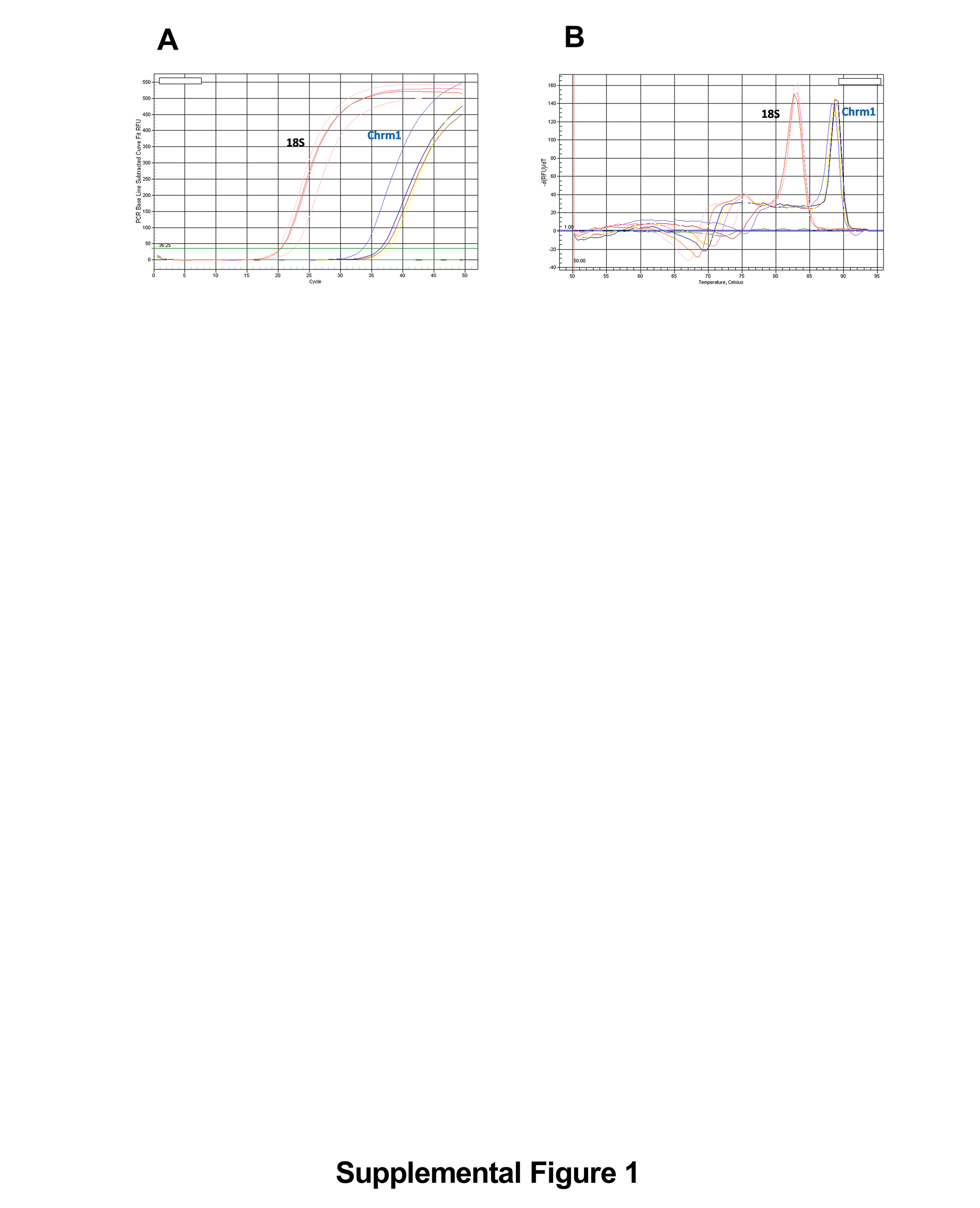

Supplement: Supplementary file 1 — Supplemental Fig. 1. M1 receptor expression in human neuroblastoma SH-SY5Y cells. M1R expression detected in SH-SY5Y cells measured by real time qPCR. Method: RNA was extracted from cultured SH-SY5Y cells using TRIzol® Reagent (Invitrogen). Complementary DNA (cDNA) was synthesized from RNA samples by using the iScript™ gDNA Clear cDNA Synthesis Kit (Bio-Rad) according to the manufacturer’s instructions. Quantitative real-time PCR (QRT-PCR) was performed using Bright Green Master mix (Abmgood Co., Richmond, Canada) compatible with the iQ5 Cycler machine (Bio-Rad). The mRNA level of 18S was used for normalization. Primer sequences for gene expression analysis are listed as follows: M1R (CHRM1)-F: 5′- CGGAACTCTGCAACAACAAAGCCTTCCG -3′, M1R (CHRM1)-R: 5′- CTTGCGCCAGCGTCTCTTGT-3′, 18S-F: 5′-GCCGCTAGAGGTGAAATTCTTG-3′, 18S-R: 5′- CATTCTTGGCAAATGCTTTCG-3′. (PNG 215 kb) [file 12035_2022_3003_Fig9_ESM.png]

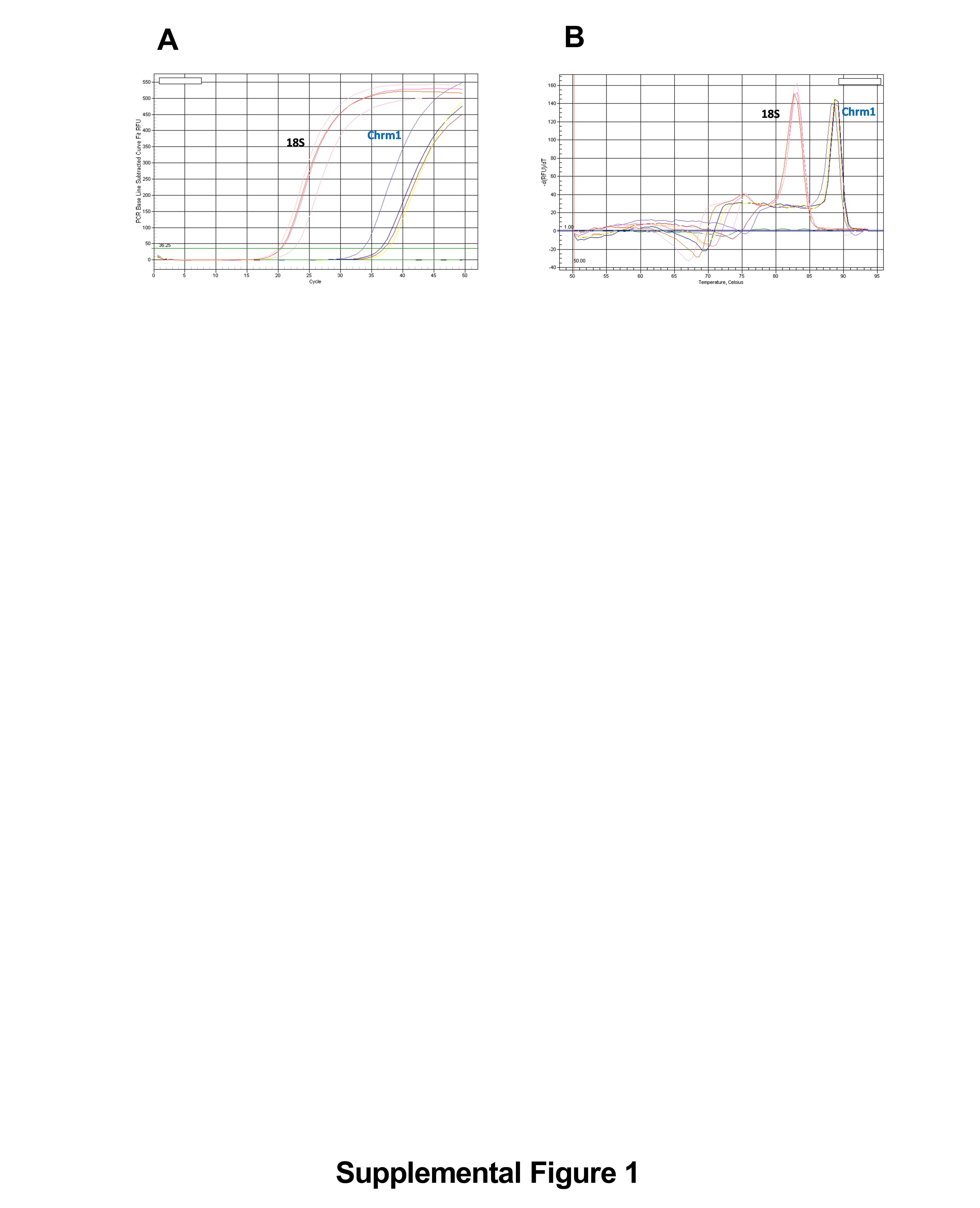

Supplement: Supplementary file 2 — High resolution image (TIF 3380 kb) [file 12035_2022_3003_MOESM1_ESM.tif]

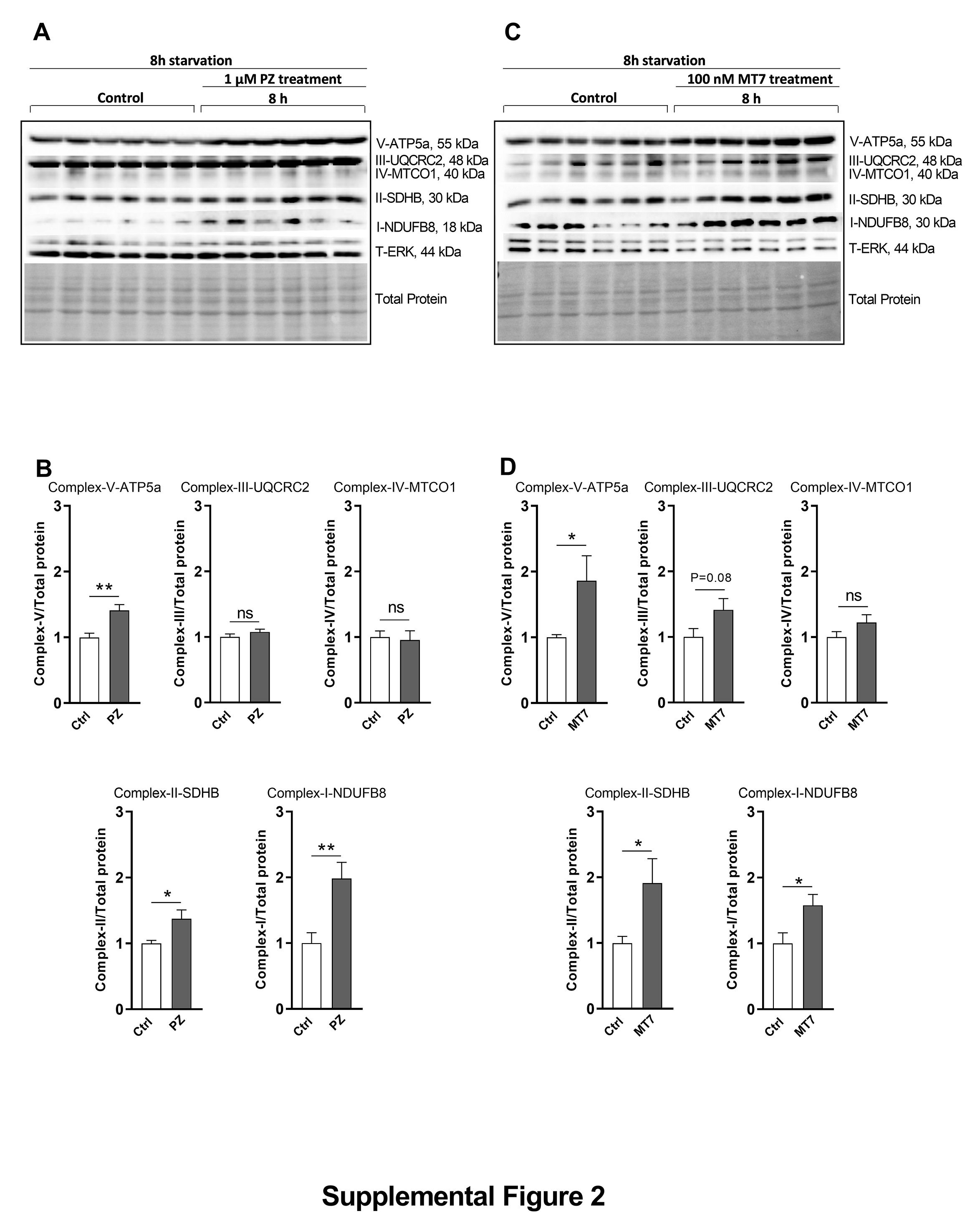

Supplement: Supplementary file 3 — Supplemental Fig. 2. Pirenzepine and MT7 treatment increase the expression of electron transport chain proteins in serum deprived conditions. A, B SH-SY5Y cells were serum deprived and treated with/without 1 μM PZ for 8h and lysates subjected to Western blotting. Starvation and treatment were started at the same time point. Specific proteins from each respiratory complex were quantified and expressed relative to total protein. C, D SH-SY5Y cells were serum deprived and treated with/without 100 nM MT7 for 8h and lysates subjected to Western blotting. Data are expressed as mean ± SEM, n = 6 replicates: *p < 0.05 or **p < 0.01 or ***p < 0.001 vs control by unpaired Student’s t-test. PZ, pirenzepine. (PNG 768 kb) [file 12035_2022_3003_Fig10_ESM.png]

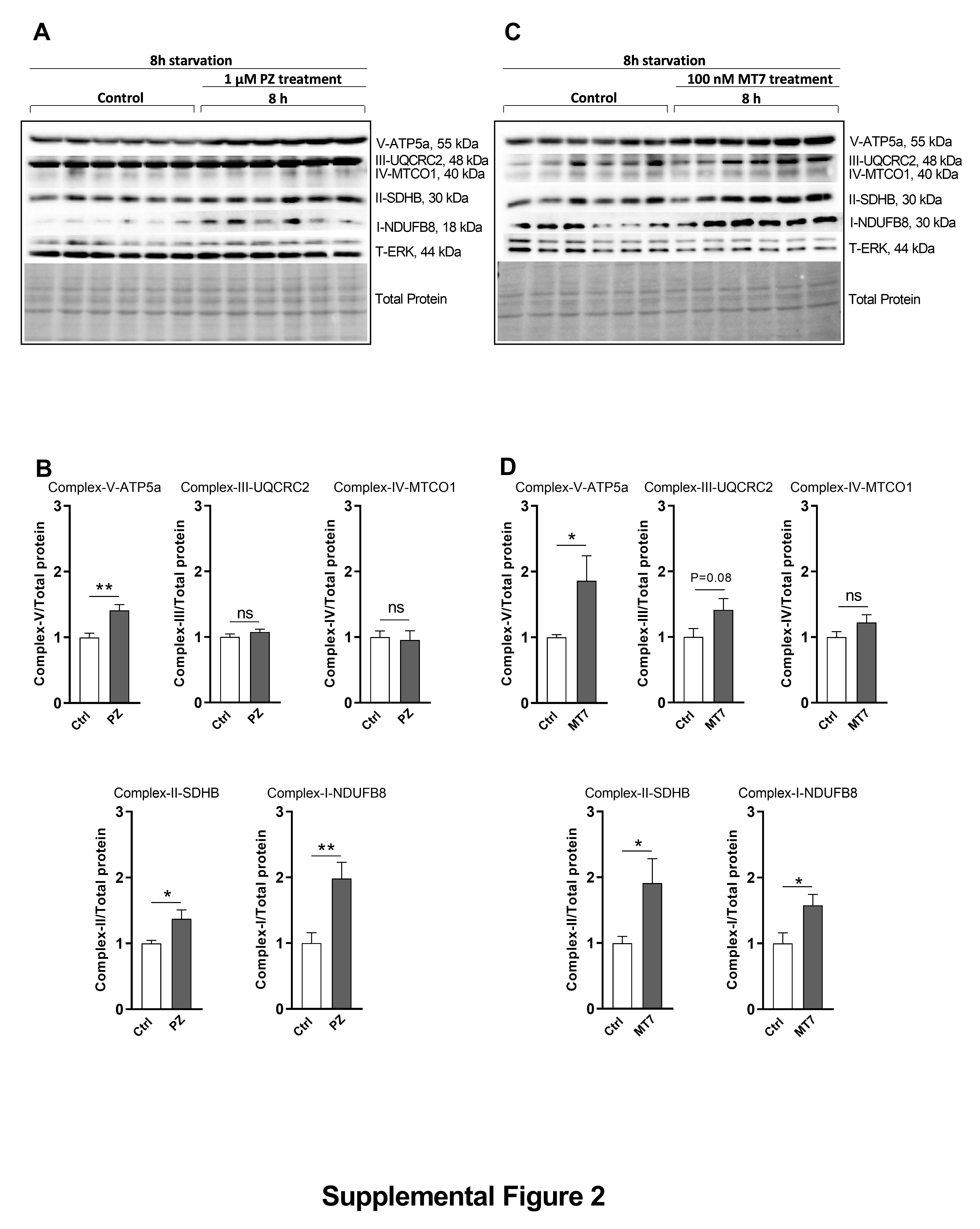

Supplement: Supplementary file 4 — High resolution image (TIF 12549 kb) [file 12035_2022_3003_MOESM2_ESM.tif]

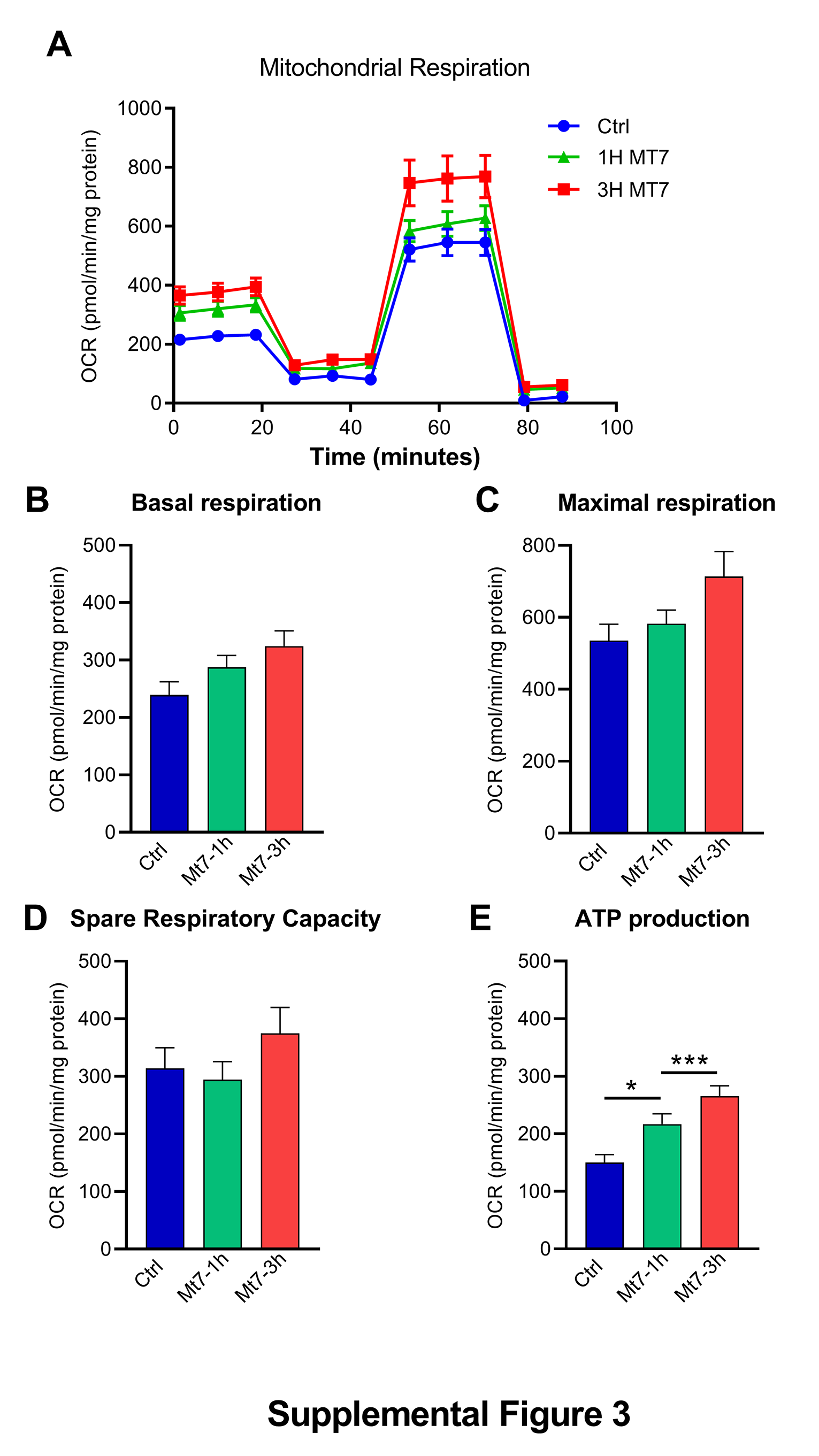

Supplement: Supplementary file 5 — Supplemental Fig. 3. M1R antagonist upregulates mitochondrial respiration and ATP production in SH-SY5Y cells. A-E. Mitochondrial respiration was measured using Seahorse XF24 Analyzer. Data were normalized to protein concentration units per well prior to statistical analysis. SH-SY5Y cells were serum deprived and treated with/without 100 nM MT7 for 1h and 3h. Data are expressed as mean ± SEM, n = 5-7 replicates; *p < 0.05 or **p < 0.01 or ***p < 0.001 by one-way ANOVA with Tukey’s post hoc test. Method: An XF24 analyzer (Seahorse Biosciences, Billerica, MA, USA) was used to measure the basal level of mitochondrial oxygen consumption rate (OCR), the maximal respiration, the spare respiratory capacity and the coupling efficiency. In short, SH-SY5Y culture medium was changed 1 h before the assay to unbuffered DMEM (Dulbecco’s modified Eagle’s medium, pH 7.4) supplemented with 1 mM sodium pyruvate, and 5 mM D-glucose. Four mitochondrial complex inhibitors including oligomycin (1 μM), FCCP (4 μM) and rotenone (1 μM) combined with antimycin A (1 μM) were injected sequentially through ports in the Seahorse Flux Pak cartridges. Oligomycin acts as an irreversible ATP synthase inhibitor, FCCP as an uncoupler, rotenone as Complex I inhibitor, and antimycin A as an inhibitor of Complex III of the mitochondrial electron transport system. After OCR measurement, cells were subjected to protein assay (DC protein assay) for normalization purposes. OCR measures from each well were normalized to total protein levels and are presented as pmoles/min/mg protein. (PNG 258 kb) [file 12035_2022_3003_Fig11_ESM.png]

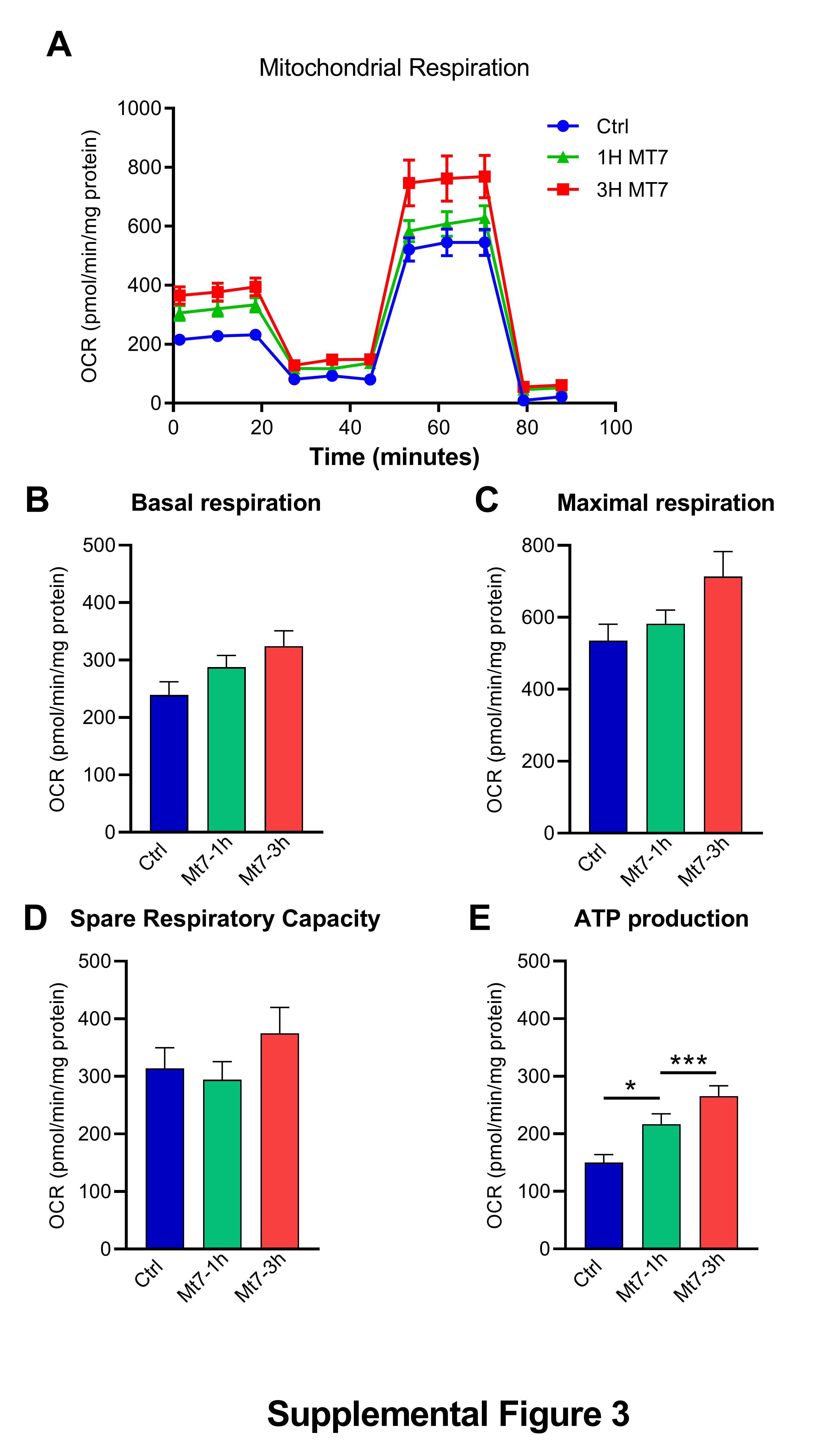

Supplement: Supplementary file 6 — High resolution image (TIF 2079 kb) [file 12035_2022_3003_MOESM3_ESM.tif]

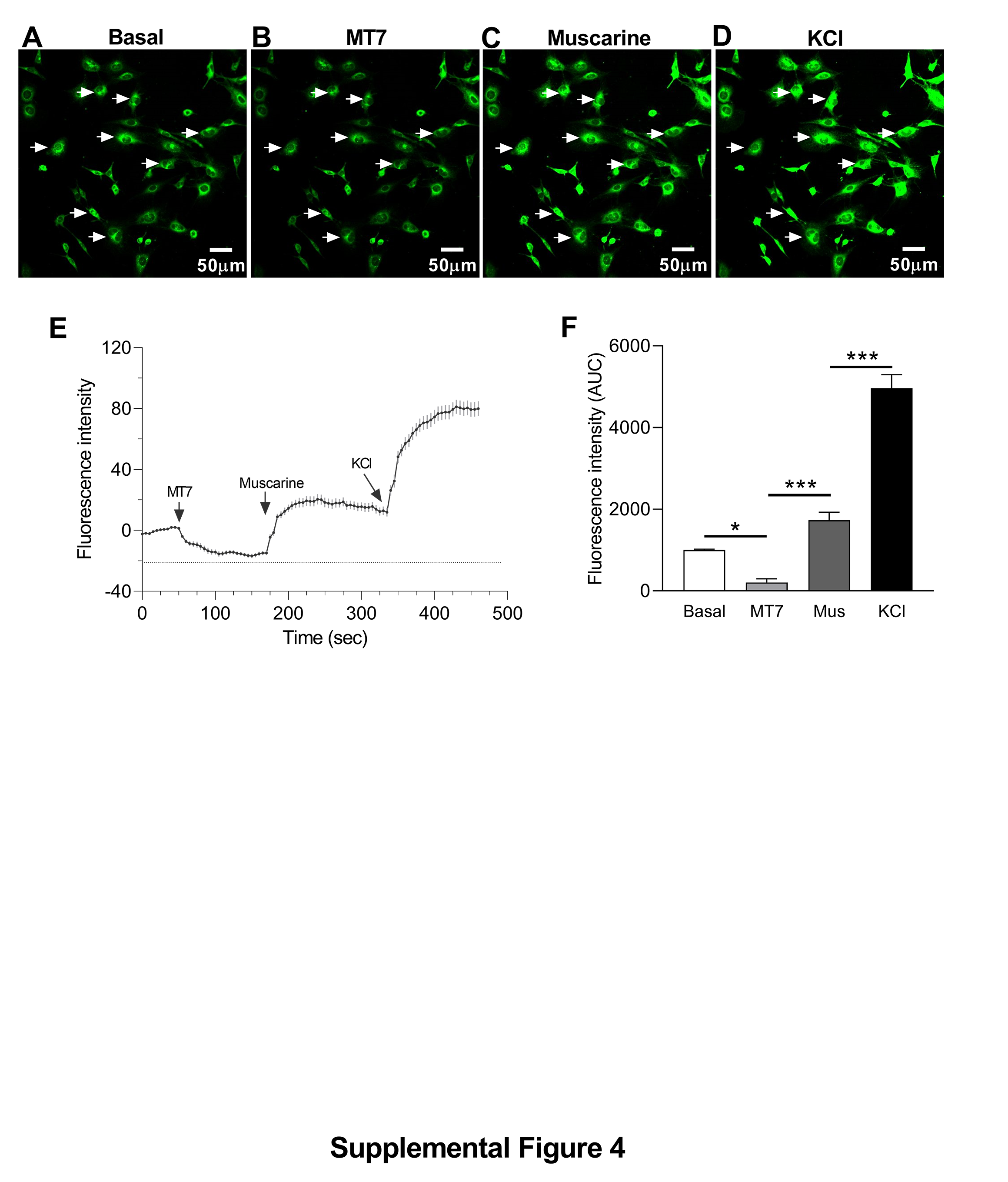

Supplement: Supplementary file 7 — Supplemental Fig. 4. Changes in the plasma membrane potential in response to M1R antagonists or agonist in SH-SY5Y cells. A-D Confocal images of cultures of SH-SY5Y cells in the presence of the voltage sensor probe DiBAC4(3) showing fluorescence at basal (A) and after administration of 100 nM MT7 (B), 100 μM muscarine (C), and 90 mM KCl (D). Arrows indicate some of the cells that responded to MT7. E-F. Traces of DiBAC4(3) fluorescence intensity (E) and AUC (F) showing the changes in plasma membrane potential measured in response to MT7 followed by muscarine and KCl. The AUC was estimated for 1 minute before each treatment (MT7, Mus, KCl) from the baseline to a fluorescence level of -20. Data are expressed as mean ± SEM, n = 38 neurons; *p < 0.05 or **p < 0.01 or ***p < 0.001 by one-way ANOVA with Tukey’s post hoc test. (PNG 898 kb) [file 12035_2022_3003_Fig12_ESM.png]

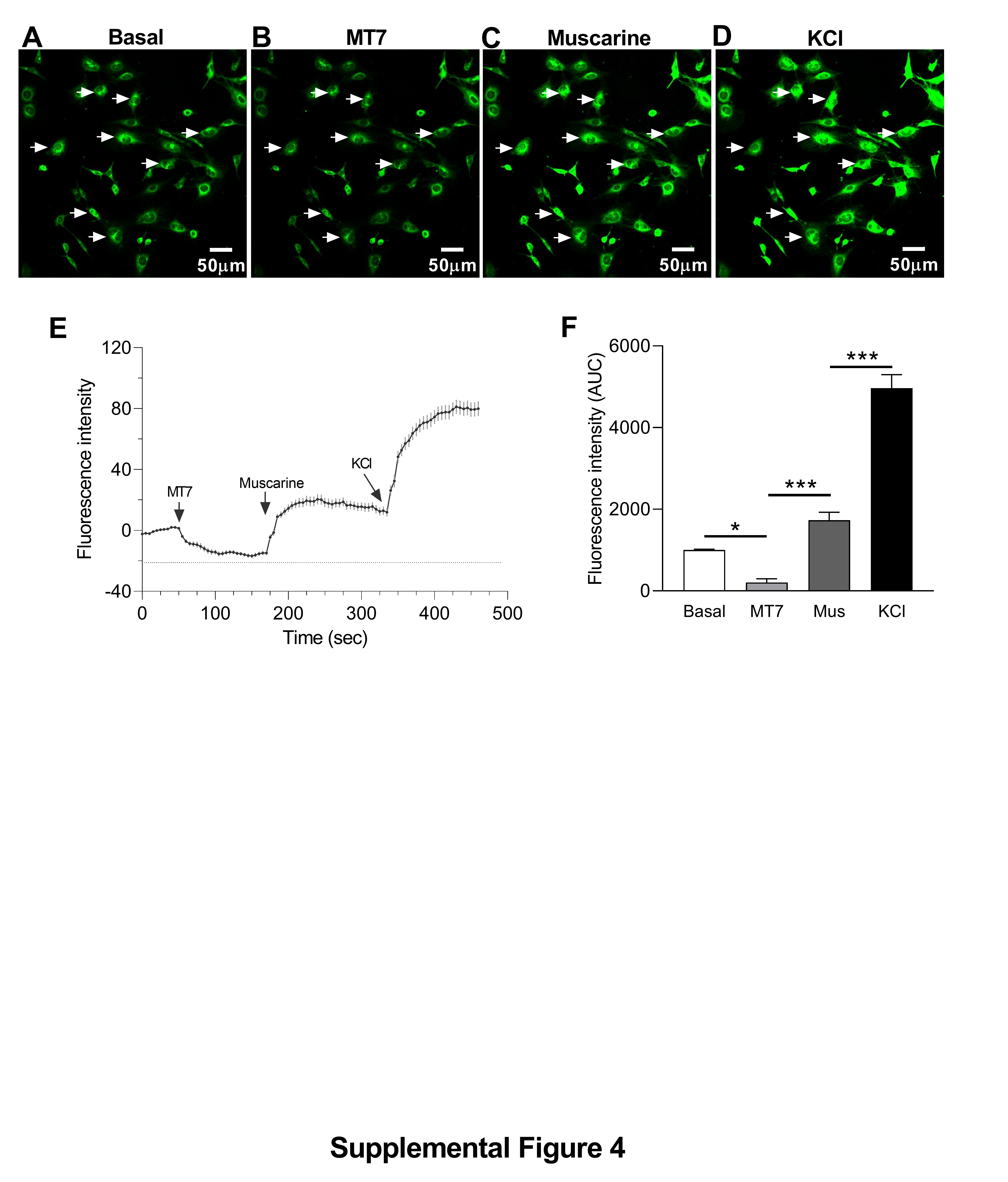

Supplement: Supplementary file 8 — High resolution image (TIF 8942 kb) [file 12035_2022_3003_MOESM4_ESM.tif]
